# Predicting late renal toxicity using a two-component repair model among pediatric patients receiving total body irradiation for stem cell transplant

**DOI:** 10.1016/j.ctro.2025.100984

**Published:** 2025-05-22

**Authors:** Eric D. Ehler, Grace H. Hutchinson, Jianling Yuan, Kathryn E. Dusenbery

**Affiliations:** University of Minnesota Department of Radiation Oncology, Minneapolis, MN 55455, USA

## Abstract

**Purpose/Objectives:**

Late renal toxicity can be impactful for stem cell transplant (SCT) patients with total body irradiation (TBI) as part of the conditioning regimen. This work describes a two component repair model to predict renal toxicity among pediatric patients.

**Materials/Methods:**

Systematic literature searches described in two other reports were used to identify articles describing late renal toxicity in children treated with TBI as part of SCT. Six studies were included with a combined sample size of 172 patients. Data pertaining to TBI and renal toxicity endpoints were extracted. Probability of renal toxicity was analyzed in relation to TBI dose, fractionation, and dose rate. A logistic regression model was developed using studies with sufficient TBI and pediatric outcome data. The toxicity was calculated from reported rates from each study and were primarily based on abnormal laboratory values.

**Results:**

Physical dose and a traditional method of calculating EQD2 were tested for a significant dose response relationship although none were found. Instead, a statistically significant relationship was found with a two component sublethal repair EQD2 calculation model using logistic regression (p = 0.03). The model predicted the odds ratio for renal toxicity to be 2.9 Gy^−1^. Predicted probabilities of 5 %, 10 %, and 50 % clinically significant renal toxicities are associated with EQD2 values of 10.4, 11.1, and 13.2 Gy, respectively.

**Conclusions:**

A model to predict the risk of renal toxicity was determined. This model indicates that dose rate and the time interval between fractions are important factors in renal toxicity for pediatric patients receiving SCT with TBI as part of the conditioning regimen.

## Highlights

• Efforts have been made recently to reduce toxicities in myeloablative TBI.
• We utilize a linear quadratic schema with two component dose rate repair mechanism.
• We compare this kidney dose response model to others in the literature.
• Dose rate is an important factor, along with total dose and dose per fraction.

## 1 Introduction

Stem cell transplant (SCT) is a vital treatment modality for various pediatric hematologic malignancies and non-malignant disorders. The preparative regimen is a key component of SCT, of which total body irradiation (TBI) can be an integral component. Chronic renal toxicity is a late radiation effect, identified as chronic kidney disease, which can have a latency lasting months to years after irradiation.[[1], [2], [3]] Various cell types and tissues within the kidney exhibit distinct reactions to radiation injury, each manifesting unique effects and temporal patterns.[3] This diversity within the organ results in a multifaceted and intricate response to irradiation. Recent works by Hoeben, et al.[1] and Poppe et al.[2] each provided a systematic review of the available literature on kidney toxicities in pediatric populations receiving TBI as part of SCT.

TBI techniques often vary considerably from institution to institution.[[4], [5], [6], [7]] Notable variations in TBI techniques are: total dose, dose fractionation, dose rate, use of blocks and/or compensators, beam energy, and beam orientation. This broad range of techniques are an important consideration for radiation dose response modeling.

Models for renal toxicity following TBI have been described previously [3,[8], [9], [10]]. In the model by Poppe et al.[2], a linear quadratic (LQ) correction was used, however, it lacked an incomplete repair component to account for differences in dose rate. Cheng et al.[8] did not utilize a LQ formalism but treated total dose, number of fractions, number of fractions per day, and dose rate as independent variables. Two studies by Kal et al.[9,10] used an LQ formalism with a dose rate correction, however, the correction used consisted of a single half-time repair value.

Two component sublethal damage repair was first suggested by Turesson and Thames[11]. Subsequently, two component repair kinetics have been observed in animal models for the lung[12,13], spinal cord[14,15], skin[16], and kidney[17]. Utilizing rodent kidney colony forming cell survival data for an array of radiation fractionation techniques, Millar et al.[17] found an alpha beta ratio (α/β) of 2.07 Gy with repair half times of 0.15 h and 5.0 h. The 0.15 h repair half time represents a “fast” short time frame repair component while the 5.0 h repair half time represents a “slow” long time frame repair component. The authors describe the relative contribution of each half time repair as “… about 34 % of the sublethally derived lethal damage being associated with the longer repair process”[17]. With the majority cell response coming from the short 0.15 hr repair component, dose rate has the potential to have a considerable impact. This work will describe a kidney dose response model, utilizing a two-component repair time with an incomplete repair correction[18], for renal toxicity among patients receiving SCT with TBI as part of the conditioning regimen.

## 2 Methods and Materials

Literature search results from Supplementary Table 3 in Hoeben et al.[1], as well as studies included in the analyses by Poppe et al.[2], Cheng et al.[8], and Kal et al.[9,10] were reviewed. Each of the studies were independently analyzed for the following data: total dose, fractionation, dose rate, time between fractions, TBI techniques such as kidney blocking, toxicity endpoint, patient demographics, and toxicity rate. Based on our review the following twelve studies, shown in Table 1, were initially identified as sufficient for the extraction of pediatric patient population outcomes as well as sufficient description of the TBI technique to calculate a LQ effective dose with incomplete repair. An additional study (study number 13) is included in Table 1. This study is the most recent analysis of outcomes from the authors’ institution and is meant to serve as an independent validation cohort.

**Table 1:** 

Study No.	Study 1st Author	Age Range (years)	N	Total Dose (Gy)	Dose per Fraction (Gy)	Dose Rate (cGy/min)	Toxicity Rate	EQD2 (Gy)	Toxicity Endpoint	Patients Included
1	Bradley [19]	2–18	36	13.2	1.20	12	0 %	11.1	Requiring dialysis or having elevated renal function tests	All patients receiving SCT with TBI
			10	13.5	1.50	12	30 %	11.8		
2	Chou [20]	1–18	58	12.0	2.00	15	3.5 %	11.8	Chronic renal insufficiency with elevated BUN & Cr or failure requiring dialysis	All patients receiving SCT with TBI
3	Freycon [21]	2–18	71	12.0	2.00	10	7.0 %	11.4	“Treated chronic renal failure”	Disease Free Survival (DFS) > 4 yr & age > 18 at time of study
4	Frisk [22]	4–18	26	7.5	7.50	15	27 %	11.9	Minimum GFR < 70, 6 months post-SCT; 5/7 recovered 90 > GFR > 70; one had GFR = 70 ten years post-SCT; one had GFR = 67 at 6 months post-SCT; one had full GFR recovery	DFS > 6 months
5	Gerstein [23]	3–16	43	11.1/12.0	1.5 – 2.0	21.7–35*	7.0 %	11.1	Increase of serum creatinine > 1.25 times age adjusted normal range; grade 2 late renal dysfunction CTCAE v3.0	Survival > 12 months
6	Künkele [24]	2–19	29	12.0	2.00	12.5** [25]	17 %	11.5	GFR < 90 at six months post-SCT, No “significant” renal impairment	Alive with follow up at the time of study; average 8 yr post-SCT (Range:2–22 yr)
7	Liesner [26]	5–14	7	9.0	9.0	7.5**[27]	14 %	12.5	Acute renal failure, partial recovery with GFR of 44	Alive at the time of study 1.5 – 9 years post-SCT with follow up
8	Lönnerholm [28]	2–18	22	7.5	7.50	15	18 %	11.9	> 35 % change in GFR at six months post-SCT with proteinuria and hematuria one had GFR < 70 at 2 + years, three had GFR < 90 at 2 + years	DFS > 6 months
9	Mulchahy Levy [29]	0–3	14	12.0	2.00	9	14 %	11.3	1 patient had renal failure requiring dialysis & transplant, 1 had renal insufficiency with low CrCl	Survive at least 1 year with follow up
10	Tarbell [30]	3-15†	5	8.5	8.50	5	20 %	11.3	Concurrent renal dysfunction and anemia between 3 – 7 months post-SCT; 3 biopsies showed significant injury (selection criteria unclear)	DFS > 3 months with follow up
			5	12.0	2.00	10	20 %	11.4		
			6	13.0	2.16	10	50 %	12.9		
			12	14.0	1.75	10	33 %	12.8		
11	Van Why [31]	0–18	39	13.2	1.65	14	21 %	12.0	Doubling of baseline Cr or Cr clearance < 50 mL/min/1.73 m^2^, one patient required dialysis, two had renal insufficiency at 5 and 8 months, 3 had proteinuria and hypertension	Survival & follow-up > 60 days
12	Watanabe Nemoto [32]	1–17	6	12.0	2.00	10	0 %	11.4	eGFR < 90 & continued to decrease	Survival > 5 years
13	Zhang [33]***	1–22	38	13.2	1.65	17	16 %	12.1	Persistent increase of serum creatinine of > 1.2 mg/dL or ≥ 1.25 times age adjusted normal range	DFS > 3 months
			79	13.2	1.65	11	10 %	11.8		

†: ages only given for pediatric patients with renal toxicity, not the whole cohort; *Averaged over combined beam arrangement technique; **dose rate data was not provided in the original article and was inferred from description of the institution’s TBI technique in cited publication, ***institutional data published as abstract only; GFR in units of mL/min/1.73 m^2^.

Table 1 demonstrates various disparities in reporting of renal toxicity, categorized into two main differences: 1) the patient population within which the toxicity rates were compared, and 2) the clinical endpoints reported. Certain reports (Studies 3, 6, 7, and 12) provided renal toxicity rates only for individuals who were still alive several years post-SCT. For instance, Study 12 solely presented data on 9 SCT patients who survived beyond 5 years (out of a total of 24 patients treated in the same time frame)[32]. Conversely, two studies (Studies 1 and 2) reported renal toxicity rates for all patients undergoing SCT[19,20]. Given the mortality rate associated with diseases treated through SCT, these variations can substantially affect the reported toxicity rate.

In the second main difference noted in Table 1, there was notable variability in how renal toxicity was determined. While some studies defined renal toxicity as kidney failure requiring intervention, others defined toxicity on abnormal lab values, primarily GFR and creatinine. These variations can substantially affect the reported toxicity rate. Ideally, toxicity grades such as those defined by the Radiation Therapy Oncology Group (RTOG)/European Organization for the Research and Treatment of Cancer (EORTC) or National Kidney Foundation (NKF) should be used for the prediction modeling. For the purpose of this paper, toxicity rate as reported by each individual study was used, as these toxicities, whether GFR below a cutoff value or kidney failure requiring dialysis, were deemed clinically significant by the authors.

Considering the issues discussed above, Studies 1, 2, 3, 6, 7, and 12 were excluded from consideration in the beam model (see Table 1) due to concern of underreporting of toxicity or inadequate follow up. The remaining 6 studies included for the modeling generally represent a patient population with disease free survival 2 – 12 months post SCT, long term follow-up, and with renal toxicity determined from laboratory testing (either GFR or serum creatinine) at some defined level either in absolute terms (e.g. GFR < 70 mL/min/1.73 m^3^) or a change from baseline. The remaining variability in the definition of renal toxicity cannot reasonably be controlled and still allow meta-analysis due to the limited number of studies and patient population sizes.

Gerstein et al.[23] provided a single institution description of renal toxicity. However, three different fractionation techniques were used. In addition, a different total dose (11.1 Gy) was used for Co-60 based TBI treatments before the introduction of medical linear accelerator based TBI (12 Gy). For this report, renal toxicity was not described for each type of TBI fractionation technique so the EQD2 was calculated for each technique and a weighted sum based on the proportion of patients receiving each fractionation technique was used to determine a single estimated mean EQD2. Tarbell et al.[30] also reported several TBI fractionation techniques including a single fraction TBI cohort, but the toxicities were reported individually for each technique. Most reports included in this analysis provided data for a diverse range of ages of pediatric patients, with the exception of one study which reported for patients all under 3 years old at the time of SCT[29].

The physical doses were converted to Equivalent Dose in 2 Gy-Fraction (EQD2) using the LQ formalism. As stated previously, the LQ formalism allows for inclusion of the effects of dose rate and incomplete repair (for multiple fraction per day regimens).[34] EQD2 was calculated with the following:

(1) $$\begin{array}{c} EQD2=D\frac{d∙C+\left(\alpha /\beta \right)}{2+\left(\alpha /\beta \right)} \end{array}$$

where D is the total dose delivered, d is the dose per fraction, and α/β is the linear-quadratic cell kill parameter. C is the parameter that accounts for incomplete repair between fractions, denoted by $H_{m}$, and repair during irradiation, g.C is calculated by:

(2) $$\begin{array}{c} C=g+2\frac{\cosh\left(\mu t\right)-1}{{\left(\mu t\right)}^{2}}∙H_{m} \end{array}$$

where:

(3) $$\begin{array}{c} g=\frac{2\left[\mu t-1+e^{-\mu t}\right]}{{\left(\mu t\right)}^{2}} \end{array}$$

(4) $$\begin{array}{c} H_{m}=\left(\frac{2}{m}\right)∙\left(\frac{\phi }{1-\phi }\right)∙\left(m-\frac{1-\phi^{m}}{1-\phi }\right) \end{array}$$

In equations (2), (3), μ is equal to the natural log of 2 divided by the repair half time ($t_{1/2})$, $\mu =\frac{ln\left(2\right)}{t_{1/2}}$; and t is the irradiation time, determined by the fraction dose divided by the dose rate. In equation (4), m is the number of fractions per day and ϕ is determined by: $\phi =e^{-\mu ∙\Delta T}$ where μ is the same as previously described and ΔT is the time interval between fractions. It should be noted that the most common form of EQD2 calculation omits the C term in equation (1), which we will hereafter refer to as the “traditional EQD2”. Traditional EQD2 was calculated for comparative purposes using an α/β of 3 Gy which is a typical value for late kidney response[34]; previous TBI-renal toxicity modelling studies used α/β values of 2.5 Gy [9,10] and 3.4 Gy[2].

In order to account for the two component sublethal damage repair, EQD2 was calculated for $t_{1/2}$ = 0.15 hr and $t_{1/2}$ = 5.0 hr separately. Then a weighted sum was calculated to determine the final EQD2 using weighting factors of 0.66 and 0.34 for $t_{1/2}$ = 0.15 hr and $t_{1/2}$ = 5.0 hr, respectively.[17].

Logistic regression was conducted using Comprehensive Meta-Analysis V3 software (Biostat, Englewood, NJ). Due to variability among studies, such as differences in patient diagnosis, demographics, TBI methods, definitions of renal toxicity, chemotherapy regimens, and GVHD management, a random effects model (Knapp-Hartung method) was chosen over a fixed effects model. The Knapp-Hartung random effects method provides a more precise estimation of error intervals for heterogeneous data.[35].

The probability, p, of renal toxicity was modeled by logistic regression:

(5) $$\begin{array}{c} p=\frac{1}{1+e^{-\left(\beta_{0}+\beta_{1}∙EQD2\right)}} \end{array}$$

where $\beta_{0}$ is a constant and $\beta_{1}$ is the coefficient of the predictor variable, EQD2. An Odds Ratio (OR) for the EQD2 can be determined by:

(6) $$\begin{array}{c} \mathrm{OR}=e^{\beta_{1}} \end{array}$$

Since the EQD2 is a continuous function, the OR value will represent the change in odds per unit change (i.e. 1 Gy) in EQD2.

## 3 Results

**Fig. 1:**
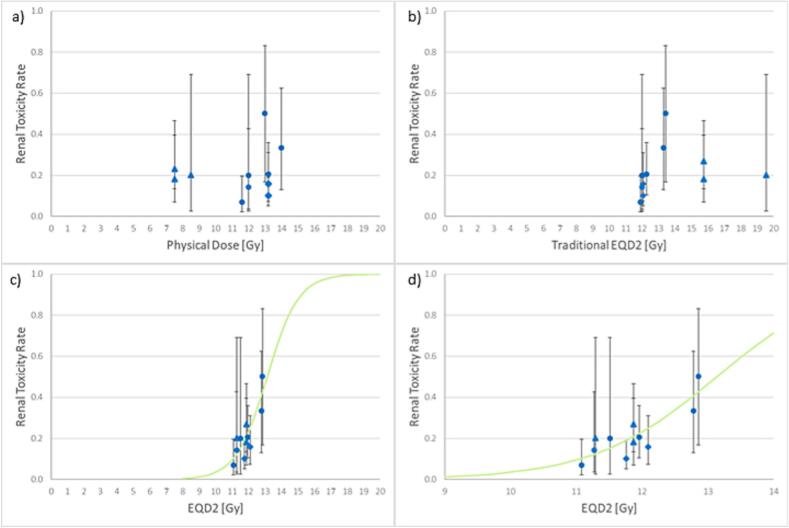
A–d. renal toxicity rates with respect to a) physical dose and b) traditional eqd2, c) eqd2 as described in the methods and materials section, d) same as in c) but focused on a narrower range on the x-axis.

The renal toxicity rates are shown in Fig. 1a-d. The triangles represent renal toxicity from studies (or patient cohorts for studies with multiple reported TBI fractionation schedules) using single fraction TBI. The circles represent fractionated TBI patient groups. Diamonds represent institutional data to serve as a validation of the model. The error bars indicate the 95 % CI. No statistically significant dose response is observed for the use of the physical dose (Fig. 1a) or the traditional EQD2 using an α/β value of 3.0 Gy (Fig. 1b). It should be noted in Fig. 1a and Fig. 1b that the single fraction TBI data is distinct from the fractionated TBI data on the x-axis with no discernable difference in the toxicity rate (y-axis). In Fig. 1c and 1d, a statistically significant response was found when utilizing an EQD2 that incorporates the two component incomplete repair model (p-value = 0.031). The logistic regression fit is shown in Fig. 1c and 1d by the solid line. Predicted probabilities of 5 %, 10 %, and 50 % clinically significant renal toxicities are associated with EQD2 values of 10.4, 11.1, and 13.2 Gy, respectively. The logistic regression coefficient, $\beta_{1}$, had a value of 1.06 (0.13 – 1.99, 95 % CI) which corresponds to an odds ratio of 2.9 Gy^−1^ (the unit Gy^−1^ is in reference to EQD2). The response model falls within the 95 % confidence intervals for the validation dataset originating from the authors’ institution. Inclusion of this data into a new model results in only a slight change in the model fit; p-value = 0.021, $\beta_{1}$ = 1.07 (0.21 – 1.94, 95 % CI).

## 4 Discussion

In Fig. 1c and 1d, it can be seen that the two component sublethal repair LQ model achieved good agreement between fractionated and single fraction TBI data. One area of interest is the interplay of the single fraction and fractionated data when comparing the physical dose, traditional EQD2, and the two-component sublethal damage EQD2 as seen if Fig. 1a, 1b, and 1c, respectively. For the physical dose (see Fig. 1a), single fraction TBI studies all report a lower dose than the total dose used in the fractionated studies. However in Fig. 1b, the single fraction TBI data results in a greater traditional EQD2 compared to fractionated TBI. When a two-component sublethal repair model is applied, the apparent discrepancies between fractionated and single fraction TBI data are reconciled, as illustrated between Fig. 1c. The longer beam on time typical of single fraction TBI (due to the high dose per fraction) and the short repair half-life component of 0.15 hr, permit substantial cellular repair during treatment, leading to a reduced EQD2 with the two-component model compared to traditional EQD2.

A recent renal toxicity meta-analysis[2] conducted as part of the Pediatric Normal Tissue Effects in Clinic (PENTEC) consortium used traditional EQD2 (α/β=3.4Gy); the study included both TBI as well as Wilm’s tumor treatment data. Our study, while employing similar datasets as the PENTEC analysis, specifically excludes data pertaining to Wilms tumor. The PENTEC study delineated models across three toxicity levels: severe, moderate, and mild. Our dataset corresponds most closely to the “moderate” toxicity category of the PENTEC work based on the description of the reported toxicity endpoints[2]. However, based on our interpretation of the “moderate” toxicity data in the PENTEC work, it appears that reports with the highest EQD2 values are those utilizing single fraction TBI[2]. For example, a single fraction TBI regimen (9 Gy x 1 fraction) reported a traditional EQD2 of 21.6 Gy in the PENTEC study, which adjusts to 12.4 Gy with the two-component sublethal repair correction EQD2 used in our model. Given these insights, reevaluating the PENTEC models using a two-component EQD2 calculation or at least analyzing fractionated and single fraction TBI data separately could yield more accurate interpretations. Finally, while the PENTEC report successfully examined severe toxicity as a more clinically significant endpoint, the application of the random effects model in this study, combined with the limited number of relevant studies and the low event frequency, precludes the attainment of a statistically significant model given the current data.

A meta-analysis series by Kal et al.[9,10], which included both pediatric and adult datasets, applied dose rate corrections using a single-component repair half-time of 0.35 hr (α/β=2.5Gy). No correction for incomplete repair between fractions was included in the model (as it is with $H_{m}$ in equations (2), (4)). The work did not specifically describe a dose response model, but a dose limit was proposed, in a different equivalent dose parameter called the Biologically Effective Dose (BED), of 16 Gy. However, the authors did not provide a statistical justification for the limit.

Another meta-analysis[8] found dose and dose rate to be significant predictors of renal toxicity without employing a biological equivalent dose model, instead using these factors as predictor variables in a multivariate analysis. This study, along with those by Kal et al.[9,10], reported renal toxicity rates without adjusting for variability in the definition of renal toxicity (e.g., asymptomatic reduction in GFR versus renal failure requiring dialysis) as the PENTEC study had done[2], highlighting a potential area for methodological enhancement in future studies.

The results from the current model are relevant for optimizing TBI delivery techniques. For example, consider a standard dose fraction TBI regimen of 2 Gy per fraction for 6 fractions delivered twice daily to a total dose of 12 Gy. With traditional EQD2 calculation, since the dose per fraction is 2 Gy, the EQD2 is equal to the physical dose regardless of the dose rate or time interval between the fractions. However, the two component sublethal repair method of EQD2 calculation can still predict differences in renal toxicity if dose rates or time intervals between fractions varied between regimens. Assuming the standard fractionation scheme above with 6 h between fractions, dose rates of 5, 10, 20, and 50 cGy per minute yield EQD2 values of 10.6, 11.4, 12.0, and 12.5 Gy, respectively. The corresponding predicted renal toxicities from this model would be 6 %, 13 %, 22 %, and 33 %, respectively. On the other hand, for a 20 cGy per minute dose rate, a 4, 6, and 8 h interval between fractions (assuming 2 fractions per day) would result in EQD2 values of 12.3, 12.0, and 11.8 Gy, with predicted renal toxicities of 28 %, 22 %, and 19 %, respectively. It should again be noted that the endpoints used to calculate renal toxicity in this model were primarily defined by decline in GFR and/or Cr values. For more severe renal toxicities such as renal failure, the risk at a given EQD2 would be expected to be lower as seen in the work by Poppe et al.[2].

TBI methods to reduce renal toxicity have been described either through the use of full or partial beam blocking[36] or the use of intensity modulated techniques[37]. However, these approaches add complexity to both treatment planning and patient localization. The finding of this study suggests that decreasing the dose rate and/or increasing the time interval between fractions can also be an effective method to mitigate renal toxicity. Given dose rate may be an important factor in kidney toxicity (as well as toxicity in other organs), the clinical implication of intensity-modulated techniques warrant careful consideration. The EQD2 values reported in this work account for the protective effects of reduced dose rate; that is, they represent the equivalent dose delivered at an instantaneous dose rate (given in 2 Gy fractions), thereby isolating the impact of rate effects. For VMAT it is important to remember that the dose and dose rate to any functional subunit of an organ can be much different from another in close proximity. In addition, tolerances of different substructures / subsystems of the kidney are not known (i.e. mean dose to the whole kidney may not be predictive of toxicity if the dose is highly variable).

A recent study of VMAT based TBI[38] found severe (grade 3 or 4) kidney injury in 17 % of patients (5 out of 29) in a non-pediatric population. Two dose rates were used, measured in Monitor Units (MU) per minute: 40 MU/min vs 100 MU/min. Renal toxicity was not stratified by dose rate in the report nor were severity scores defined. Notably, the reported mean kidney dose ranged from approximately 9.3 to 10 Gy. Based on our model, and estimating the actual dose rates to the kidneys to be up to 50 cGy/min, the predicted rate of toxicity would be under 3%; since the toxicities in our analysis are classified as moderate, the expected rate for severe toxicity would be even lower. It is unclear what the cause of increased rate of renal toxicity could be in the VMAT study but we point out that the study did not have a pediatric population. It also reinforces the points made in the previous paragraph highlighting the differences that arise between VMAT and traditional TBI.

A meta-analysis of lung toxicity in pediatric populations[7] found that the risk of idiopathic pneumonitis syndrome could also be reduced with the use of a lower dose rate. The study used an EQD2 with a single component repair model (i.e. with only a dose rate correction). However, the *meta*-analysis was limited to fractionated TBI data. A two component EQD2, such as in this work, should be considered for the lung (or any other organs) if both single fraction and fractionated TBI data will be used.

## 5 Conclusions

A renal toxicity model is described for pediatric patients receiving TBI as part of a SCT conditioning regimen. The dose response model uses a two component sublethal repair method to calculate an EQD2 with dose rate and time between fraction corrections. The model can inform TBI technique to mitigate late renal complications.

## Funding statement

This work has not been supported.

## Declaration of Competing Interest

The authors declare that they have no known competing financial interests or personal relationships that could have appeared to influence the work reported in this paper.
